# Vitamin E supplementation in diet ameliorates growth of Nile tilapia by upgrading muscle health

**DOI:** 10.1016/j.sjbs.2023.103558

**Published:** 2023-01-13

**Authors:** Md. Fazle Rohani, Tamanna Tarin, Jabed Hasan, S.M. Majharul Islam, Md. Shahjahan

**Affiliations:** a Laboratory of Fish Ecophysiology, Department of Fisheries Management, Bangladesh Agricultural University, Mymensingh 2202, Bangladesh; b Department of Aquaculture, Bangladesh Agricultural University, Mymensingh 2202, Bangladesh

**Keywords:** Aquaculture, Feed, Micro-nutrient, Hematology, *O. niloticus*

## Abstract

Vitamin E (VE), an important lipid-soluble antioxidant, has great influence on growth and maintenance in animal. The effects of VE supplemented diet on growth and feed usage in Nile tilapia (*Oreochromis niloticus*) was investigated in this study. Three formulated diets containing VE (0, 50 and 100 mg/kg) were fed to Nile tilapia (3.56 ± 0.16 g) in glass aquaria maintaining three replicate groups for 56 days (8 weeks). Survival, growth performance including weight gain, percent weight gain, and specific growth rate (WG, % WG, and SGR), and feed utilization comprising protein efficiency ratio and feed conversion ratio (PER and FCR) were calculated. Hemato-biochemical indices including hemoglobin level (Hb), white blood cell (WBC), red blood cell (RBC) and glucose level were analyzed. In addition, muscle morphology was examined after completion of the experiment. At the end of the trial, WG, %WG, SGR, FCR and PER increased significantly which had dietary VE supplimentation. However, no distinct changes were observed in Hb level, RBC count, WBC count and glucose level among these different dietary groups. Dietary VE treatments significantly upgraded the muscle fiber diameter and lowered the intra-muscle gap. Moreover, quantity of hyperplastic muscle fiber as well as nucleus also significantly enhanced by VE. Morphological structure of muscle characterized by a huge proportion of hyperplastic muscle that may be supposed to contribute the enhanced growth of Nile tilapia receiving VE supplemented diet. Therefore these results suggested that VE incorporation into the feed can be effective to improve the feed efficiency and maximize the growth of *O. niloticus*.

## 1 Introduction

Good quality and inexpensive feed is one of the prerequisites to a profitable and successful aquaculture (Singha et al., 2021, Rohani et al., 2022b). Feed quality not only influence the overall production but also has a very intimate relationship with the associated water quality and hence has a significant impact on the well-being of the farmed species (Guo et al., 2019, Hossain et al., 2020a, Hossain et al., 2020b, Kong et al., 2020, Uddin et al., 2007). The quality of feed largely depends on how effectively the essential nutrients are balanced in that feed and therefore feed manufacturers must pay special attention in this regards (Jahan et al., 2021, Mohamed et al., 2021). Nowadays, production of well-balanced and cost effective feed has become a great challenge all over the world because of the ceiling price of the feed ingredients (Kok et al., 2020, Singha et al., 2021). To mitigate this challenge and to ensure the production of high quality cost-effective feed, provision of several micronutrients may enhance the feed efficiency as well as feed utilization which may lower the cost associated with feed (Akter et al., 2021, Rohani et al., 2022a). Vitamins and minerals categorized as micronutrients are the essential components of animal feed for maintaining health and improving immunity.

Micronutrients refer to the essential components of feed that are required very small quantities. Micronutrients influence biochemical, metabolic and physiological processes of animal and positively improve the growth, production and immunity (Upadhaya and Kim, 2020). Micro-nutrients play a significant role in the production of farmed aquatic species (Hamre, 2011, Taslima et al., 2022). Vitamin and minerals are important micronutrients that must be provided through the diet as animal cannot able to synthesize them within the body. Various studies demonstrated the positive effects of several micronutrients (vitamin and minerals) in terms of growth, reproductive activities and immunity of animals (Defoirdt et al., 2011, Hayat et al., 2010). Vitamin E (VE), act as an antioxidant and therefore prevents the oxidation process of essential fatty acids (Chen et al., 2013, Zhou et al., 2013). It was observed that dietary VE supplementation significantly improved the production of several speciesc including yellow catfish, *Pelteobagrus fulvidraco* (Lu et al., 2016), hybrid snakehead, *Channa argus* × *C. maculata* (Zhao et al., 2018), and black sea bream, *Acanthopagrus schlegeli* (Peng et al., 2009). Similarly, dietary VE significantly improved the immunity and disease resistance in Japanese flounder, *Paralichthys olivaceus* (Gao et al., 2014) and Indian major carp rohu, *Labeo rohita* (Sahoo and Mukherjee, 2002). Several studies demonstrated that deficiency of VE hampered the growth performance of sea bream, *Sparus aurata* (Tocher et al., 2002), black sea bream, *A. schlegeli* (Peng et al., 2009) and spotted snakehead, *Channa punctatus* (Abdel-Hamteid et al., 2012). Moreover, deficiency of VE exhibited several disorders such as hemoglobin breakdown, edema and muscular dystrophy in fishes (McDowell, 2000, Mourente et al., 2007). VE plays a vital role for maintaining the normal physiological process and metabolism in fish. Deficiency of VE inhibited the antioxidant capacity, fat metabolism and immunity of genetically modified farmed tilapia fingerlings (Qiang et al., 2019). VE reduced lipid peroxidation in muscle and increased glutathione level in liver of hybrid tilapia juvenile (Huang and Huang, 2004). Furthermore, VE significantly enhanced the antioxidant capacity as well as influenced the lens cortical membrane structure of tilapia (Huang et al., 2003). VE exhibited the protective role against the potent hepatotoxins (microcystin)-induced oxidative stress (Prieto et al., 2008) and decreased the toxicity resulting from a fungicide, copperoxychloride, on Nile tilapia (Hassaan et al., 2014). Moreover, VE altered the blood physiological parameters and ensured protection during stressed conditions of Nile tilapia (Ispir et al., 2011).

Nile tilapia (*Oreochromis niloticus*) is considered as the global aquatic chicken due to its world-wide distribution and it’s huge production brought it as the second most contributory species in aquaculture (FAO, 2018, Rahman et al., 2021). This species has become very popular all over the world due to some of its favorable features for instant tranquil seed production (Barman and Little, 2011), high responsive to the formulated commercial feeds (Ogello et al., 2014), short production cycle (Francis and Esa, 2016), greater edible portion with less intramuscular bones (Moesch et al., 2016) and capability to cope up with adverse environmental conditions (Chaput et al., 2020, Foysal et al., 2020). Although numerous studies confirmed that VE enhanced the growth, immunity and disease tolerance of Nile tilapia (Huang and Huang, 2004, Ispir et al., 2011, Qiang et al., 2019) but none of those clarify the role of VE on morphological alteration of muscle of Nile tilapia. Hence, the effects of VE supplementation on growth performance, hemato-biocamical parameters, feed utilization efficiency and muscle structure of Nile tilapia was investigated in this study.

## 2 Materials and methodology

### 2.1 Experimental fish

To conduct this experiment, *O*. *niloticus* fingerlings were procured from Reliance Aqua Farm, Mymensingh and stocked in the Laboratory of Fish Ecophysiology, Bangladesh Agricultural University, Mymensingh, Bangladesh. Initial mean length and weight of the collected fingerlings were 5.6 ± 0.21 cm and 3.56 ± 0.16 g, respectively. In the laboratory, fish were kept in glass aquarium having a volume of 100 L to acclimatize with the culture environment. During the acclimatization period (15 days), commercial feed was provided to the fingerlings at a rate of 5 % body weight and twice in 24 h.

### 2.2 Diet preparation

Formulation of diets for this experiment were done according to Pearson’s Square Method (Wagner and Stanton, 2012). In this experiment, three experimental diets having VE (50 mg/kg), a diet with VE (100 mg/kg) and diet with no VE (control) were prepared. Based on the findings of Shiau & Shiau (2001) and (Huang and Huang, 2004), the doses of VE in the experimental diets were fixed. At first, a hammer miller (Model DX-30B, China) was used to grind the feed ingredients into fine particles. After grinding, the fine particles of the ingredients were weighed carefully. Then, the weighed ingredients were transferred to a pre-cleaned drum mixer (SYTH-0.2, China) where these ingredients were properly mixed with molasses (binders). Analytical grade Acivit-E [Alpha Tocopheryl Acetate 20 %] (*ACI* Limited, Bangladesh) was used as a source of dietary VE in this study. For dough preparation, water was added into the bowl containing all other ingredients. Then, extrusion was done by using a pellet machine (Model DGP70-II, China). In this experiment, we prepared feed having particle size of 1.5 mm. The extruded diet particles then fully air dried and packed in sealed polythene before starting the experiment. The chemical/percentage analysis of the formulated feeds (Table 1) were carried out in the Laboratory of Fish Nutrition, Bangladesh Agricultural University, Mymensingh followed by AOAC (2005). The final proportions of VE in the diets were determined by a high performance liquid chromatography (HPLC) and the level of dietary VE demonstrated in Table 1.

**Table 1:** Experimental diets and their proximate composition based on percent (%) dry matter.

Ingredients	Diet-1	Diet-2	Diet-3
Fish meal	45	45	45
Soybean meal	25	25	25
Mustard oil cake	15	15	15
Wheat bran	10	10	10
Molasses	3	3	3
Vitamin and mineral premix (vitamin E free)^a^	2	2	2
Vitamin E (mg/kg)	–	50	100

Proximate composition (%)			
Crude protein	38.28	38.17	38.11
Crude lipid	5.05	5.78	5.36
Ash	12.71	12.86	12.65
Crude fiber	4.14	4.16	3.96
NFE	29.06	28.80	29.27
Moisture	10.73	10.23	10.81
VE (mg/kg)	ND	51.00 ± 0.11	103.00 ± 0.14

ND; not detected, ^a^ Composition of premix (per kg): Composition of premix (per kg): retinol: 50,000 IU; thiamine: 12 mg; riboflavin: 25 mg; pantothenic acid: 200 mg; pyridoxine: 15 mg; biotin: 12 mg; cyanocobalamin: 0.04 mg; folic acid: 86 mg; ascorbic acid: 120 mg; cholecalciferol: 10,000 IU; phylloquinone: 10 mg; inositol: 330 mg; zinc: 4.0 g; iron: 80 gm; manganese: 15.3 mg; copper: 427 mg; calcium: 47 gm; iodine: 2 gm; selenium 42 mg; cobalt 1.3 mg; magnesium: 100 mg; sodium chloride: 20 gm.

### 2.3 Feeding trial

To conduct the eight-week experiment, previously acclimated fingerlings (twenty in each aquarium) were randomly transferred into nine glass aquaria and divided into three dietary treatment groups and each dietary treatment groups were triplicated. The fingerlings were fed two times (9 am and 5 pm) in a day at 5 % body weight. The desired level of dissolved oxygen was ensured by provision of uninterrupted aeration system throughout the experiment. The uneaten feed and feces were removed regularly through siphoning to keep the water quality good.

### 2.4 Survival, growth and nutrient utilization indices

At the end of the trial period, the total number of trialed fish in each tank was counted and weighed carefully and documented as well. Using the recorded data, growth parameters like weight gain (WG), percent weight gain (% WG) and specific growth rate (SGR) were determined. Feed consumption indices like feed conversion ratio (FCR) and protein efficiency ratio (PER) were also calculated according to (Islam et al., 2021).

### 2.5 Proximate composition of experimental fish

Proximate composition of the experimented fish taken from each dietary treatment group was carried out according to AOAC (2005). Briefly, moisture content was determined by drying a constant amount of samples in an oven at 105⁰C and for ash in a muffle furnace at 550⁰C. For crude protein determination, amount of nitrogen in the samples were estimated through micro-Kjeldahl process at first and then multiplied by 6.25 (conversion factor). Crude fibre was determined through Fibre Tech apparatus (Tulin equipment, India). Ether extraction methods using Soxhlet apparatus was deployed for crude lipid content determination of the experimental samples.

### 2.6 Hemato-biochemical parameters

After 8 weeks, fish were (n = 6) randomly collected from each treatments and sacrificed. A heparinized plastic syringe was used to collect blood from the sacrificed fish. In the meantime, fish muscle samples were collected, stored (Bouin’s fluid), fixed and preserved (70 % alcohol, 4⁰C) until histological activities performed. Blood biochemical parameters including blood glucose (BG), haemoglobin (Hb), white blood cell (WBC) and red blood cell (RBC) were measured according to Islam et al. (2021).

### 2.7 Histological examination of fish muscle

Histological examination of the muscle structure was carried out by following the procedures describe by Rohani et al. (2021). A photomicroscope fitted with a camera (AmScope 1000) was used to capture photomicrograph of the histo-morphological alterations resulted from different dietary treatments and at the same time hyperplastic fibre was counted. Hyperplastic muscle fibres are those whose diameter<20 μm (Rowlerson et al., 1995). Nonparametric statistical techniques was applied to analyse muscle fibre diameter (Johnston et al., 1999).

### 2.8 Statistical analysis

Prior to statistical analysis, all the relevant data were tested for the variances of normality and homogeneity. Following, one way analysis of variance (ANOVA) was deployed to find out the statistically significant (p < 0.05) level among the different treatments. All these statistical analysis were carried out using SPSS software (IBM SPSS statistics, Version 22).

## 3 Results

### 3.1 Survival, growth influence and feed utilization

At the end of the experiment, the survival of the experimental fish was quite high (98–100 %), that were not influenced by VE supplemented diets (Table 2). Dietary VE considerably (p < 0.05) improved the growth indices such as WG, % WG and SGR of the fish compared to control diet. Similarly, feed usage indicators (FCR and PER) were significantly (p < 0.05) developed without considerable difference was observed in case of control group (Table 2).

**Table 2:** Growth performances of Nile tilapia fed with experimental diets for 8 weeks.

Growth Parameters	Treatments		
	Control	VE (50 mg/kg)	VE (100 mg/kg)
Initial BW (g)	3.56 ± 0.16	3.57 ± 0.16	3.56 ± 0.16
Final BW (g)	13.02 ± 1.66^a^	20.25 ± 1.74^b^	22.61 ± 1.58^b^
Weight gain (g)	9.46 ± 1.84^a^	16.68 ± 1.75^b^	19.05 ± 1.67^b^
% Weight gain	265.73 ± 4.04^a^	467.23 ± 5.22^b^	535.11 ± 7.29^b^
SGR (% / day)	1.01 ± 0.41^a^	1.35 ± 0.22^b^	1.43 ± 0.16^b^
PER	1.82 ± 0.18^a^	2.37 ± 0.33^b^	2.40 ± 0.27^b^
FCR	1.43 ± 0.07^b^	1.10 ± 0.05^a^	1.09 ± 0.08^a^
Survival (%)	98.00 ± 0.00	100.00 ± 0.00	99.00 ± 0.00

BW; body weight, FCR; feed conversion ratio, PER; protein efficiency ratio: SGR, specific growth rate. Values with different alphabetical superscripts in a row differ significantly (p < 0.05) among different diets. All values are expressed as mean ± SD.

### 3.2 Proximate composition of fish

The proximate composition analysis of the whole fish carcass of different dietary treatments was done at the end of the experiment. The results revealed that dietary treatments did not significantly (p < 0.05) affect the proximate composition of fish carcass (Table 3).

**Table 3:** Proximate composition of Nile tilapia fed with experimental diets for 8 weeks.

Proximate composition (%)	Treatments		
	Control	VE (50 mg/kg)	VE (100 mg/kg)
Moisture	76.03 ± 0.17	75.77 ± 0.13	75.85 ± 0.09
Crude lipid	2.89 ± 0.12	2.77 ± 0.15	2.74 ± 0.08
Crude protein	14.47 ± 0.08	14.49 ± 0.08	14.97 ± 0.12
Ash	2.85 ± 0.10	2.91 ± 0.09	2.85 ± 0.06
Crude fiber	1.47 ± 0.07	1.72 ± 0.10	1.56 ± 0.07
Carbohydrate	2.29 ± 0.48	2.34 ± 0.50	2.04 ± 0.37

### 3.3 Hematological parameters

Hb (g/dL) level, RBC (×10^6^/mm^3^) and WBC (×10^3^/mm^3^) count in different dietary treatments were demonstrated in Table 4. However, there was no significant (p < 0.05) difference among the treatment groups on hematological indices (Table 4).

**Table 4:** Hemato-biochemical parameters of Nile tilapia fed with experimental diets for 8 weeks.

Blood parameters	Treatments		
	Control	VE (50 mg/kg)	VE (100 mg/kg)
Hb (g/dl)	9.30 ± 0.70	11.20 ± 0.61	10.33 ± 0.07
RBC (×10^6^/mm^3^)	2.58 ± 0.05	2.67 ± 0.07	2.71 ± 0.11
WBC (×10^3^/mm^3^)	1.50 ± 0.13	1.56 ± 0.15	1.52 ± 0.11
Glucose (mg/dl)	141.30 ± 2.1	149.12 ± 2.3	158.53 ± 2.6

### 3.4 Biochemical parameters

The levels of BG were varied between 141.30 and 158.53. VE provision did not show significant (p < 0.05) influence on the BG levels of fish (Table 4).

### 3.5 Histo-morphology of the muscle

**Fig. 1:**
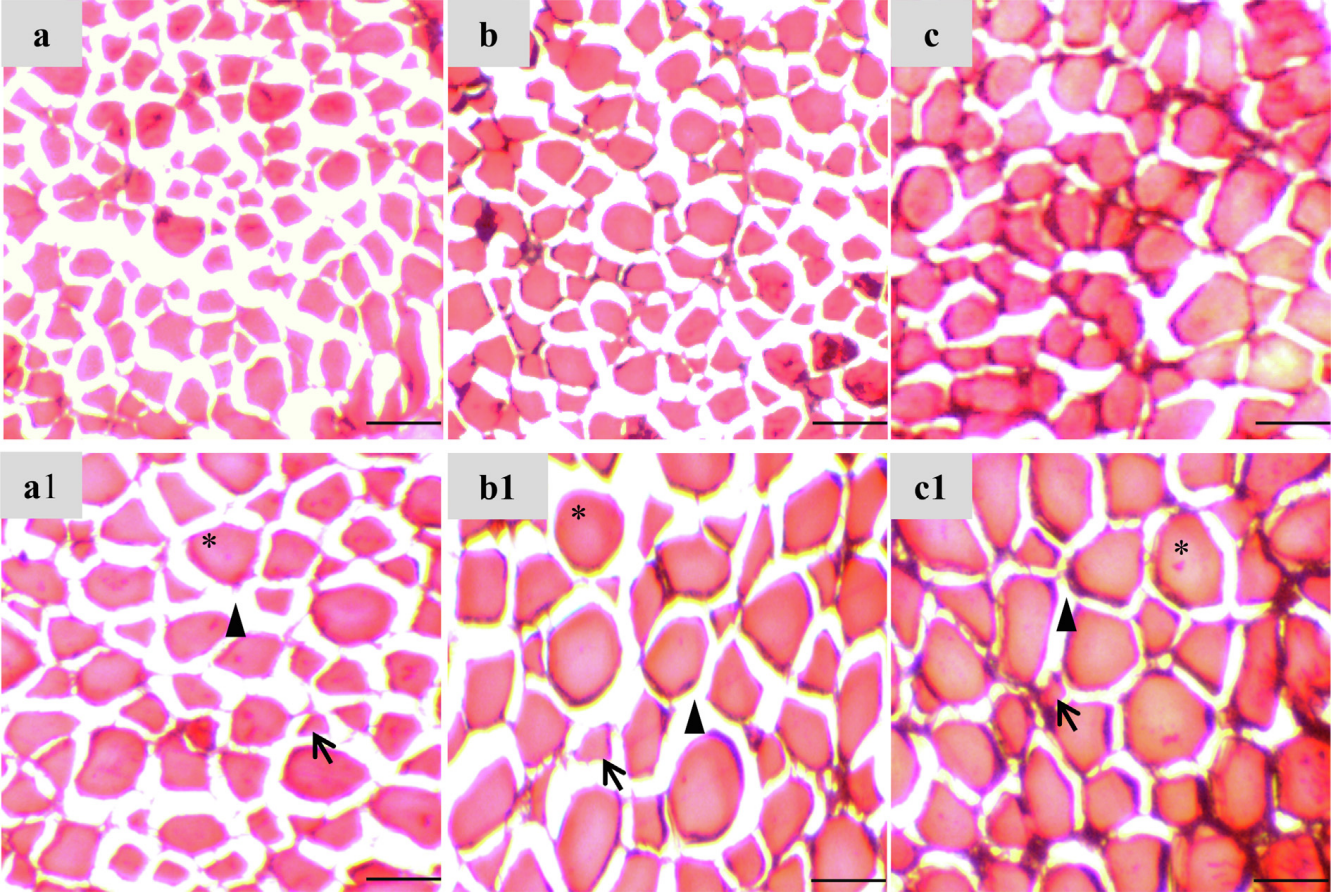
Histological changes in white muscle of Nile tilapia (*Oreochromis niloticus*) fed with different experimental diets; a & a1- Control, b & b1- VE (50 mg/kg) and c & c1- VE (100 mg/kg). Muscle nucleus indicated by an asterisk (*), arrowhead (▲) indicated fiber distance and arrow (↑) indicated hyperplastic muscle fiber. Magnification: Upper (a, b & c) panel 4X and lower (a1, b1 & c1) panel 10X. Scale bar: 100 μm.

Muscle morphology of fish treated with several treatments is demonstrated in Fig. 1. Muscle fiber diameter and distance within the muscle fiber varied from 22.15 to 37.40 μm and 5.50 to 11.50 μm, respectively. Quantity of nucleus and hyperplastic muscle ranged from 5.00 to 21.67 and 1.67 to 6.33, respectively (Table 5). Significant (p < 0.05) improvements in muscle fiber diameter, muscle fiber distance, and nucleus and hyperplastic muscle fiber number were noticed as a result of dietary VE supplementation.

**Table 5:** Changes in muscle morphology of Nile tilapia fed with experimental diets for 8 weeks.

Parameters	Treatments		
	Control	VE (50 mg/kg)	VE (100 mg/kg)
Diameter of muscle fiber (µm)	22.15 ± 2.98^a^	35.20 ± 4.15^b^	37.40 ± 5.61^b^
Distance of muscle fiber (µm)	11.50 ± 3.63^b^	8.40 ± 3.21^ab^	5.50 ± 2.94^a^
Number of nucleus	5.00 ± 1.78^a^	11.33 ± 1.36^b^	21.67 ± 3.68^b^
Hyperplastic muscle fiber	1.67 ± 1.36^a^	5.66 ± 1.36^b^	6.33 ± 0.51^b^

Values with different alphabetical superscripts in a row differ significantly (p < 0.05) among different diets. All values are expressed as mean ± SD.

## 4 Discussion

Fish is unable to synthesize VE within their body and must rely on dietary supplementation of VE (Li et al., 2018, Peng et al., 2009). VE significantly influenced the biochemical and physiological properties of fish (Chen et al., 2013, Zhou et al., 2013). In this experiment, dietary VE supplementation in diets significantly improved the growth parameters of Nile tilapia. VE averts rancidity of unsaturated fatty acids of diets as well as fish tissues and helps in maintaining normal metabolic activities, which improve feed utilization (Wassef et al., 2001). In the current study, dietary VE lowered the FCR, which is a key indicator to evaluate the quality of feed. Dietary supplementation of VE improved the feed efficiency of soft–shell turtle, *Pelodiscus sinensis* (Huang and Lin, 2004), which is consistent with our results. Several studies revealed that VE supplementation through diet improved the production and feed efficiency of parrot fish *Oplegnathus fasciatus* (Galaz et al., 2010), Rainbow trout *Oncorhynchus mykiss* (Kelestemur et al., 2012), zebrafish *Danio rerio* (Mehrad et al., 2012), yellow catfish *Pelteobagrus fulvidraco* (Lu et al., 2016), turbot *Scophthalmus maximus* (Niu et al., 2014), channel catfish *Ictalurus punctatus* (He et al., 2017) and largemouth bass *Micropterus salmoides* (Li et al., 2018). The growth improvement could be a reflection of better utilization of essential nutrients, which may be attributed to enhance intestinal height and mucosal thickness (He et al., 2017), stimulate the digestive and absorptive capability of host fish (Farhangi and Carter, 2001). The intestine has been considered an important organ of fish that has a direct relationship with digestion, absorption and transportation of nutrients. Intestinal fold height and mucosal membrane thickness influence the digestive and absorptive ability of fish (Farhangi and Carter, 2001). VE significantly promoted gut development through enhancing intestinal folds as well as mucosal membrane thickness, thus improving the digestive and absorptive capacity of fish and contributing to the growth enhancement of fish (He et al., 2017). Besides, microbial scenario of gut is considered as one of the most important index of health status (Butel, 2014, Koch et al., 2018) which is significantly influenced by various types of fish feed additives (Tapia-Paniagua et al., 2019, Almeida et al., 2021, Domínguez-Maqueda et al., 2021, Cerezo et al., 2022). Acosta et al. (2022) reported that dietary vitamin supplementation significantly enhanced the abundance of several taxa of gut microbiota of the host species that influence several important functions including digestion as well as absorption of essential nutrients and hence boost up the immune response. However, the role of dietary VE on gut microbiome of Nile tilapia warrants further investigation. Moreover, dietary VE enhanced the availability and action of enzymes associated with digestion (protease, lipase), resulting in epithelial cell proliferation and absorbency capacity of the intestine and ensuring better feed utilization and growth (Cuvier-Péres and Kestemont, 2001). In addition, dietary VE significantly influenced the secretion of antioxidant enzymes (SOD and CAT) in fish (Li et al., 2014, Wu et al., 2017), which indicated that VE might reduce fish muscle lipid peroxidation and could maintain muscle structural integrity by reducing oxidative damages. Nevertheless, active role of the dietary VE on anti-oxidative activities needs further investigation. On the other hand, VE deficiency negatively affect the growth performance of black sea bream *Arremon schlegeli* (Peng et al., 2009), spotted snakehead *C. punctatus* (Abdel-Hamteid et al., 2012), cobia *Rachycentron canadum* (Zhou et al., 2013), grass carp *Ctenopharyngodon idellus* (Li et al., 2014) and yellow catfish *P. fulvidraco* (Lu et al., 2016). These results support the positive roles of VE. Therefore, VE is recommended for better growth and nutrient utilization of Nile tilapia. On the contrary, many authors revealed no positive effect in some fish growth including golden shiner, *Notemigonus crysoleucas* (Chen et al., 2004), rainbow trout, *Oncorhynchus mykiss* (Kiron et al., 2004), pacu, *Piaractus mesopotamicus* (Belo et al., 2005), sunshine bass, *Morone chrysops* × *M. saxatilis* (Trushenski and Kohler, 2007) and meager, Argyrosomus regius (Lozano et al., 2017). Sources, types, doses as well as purity of VE in association with study period, environmental variables and responsiveness of the fish species may be responsible for these variations.

Physiological state, immunity and well-being of fish are determined by hemato-biochemical indices (Ashaf-Ud-Doulah et al., 2020, Islam et al., 2020a, Islam et al., 2020b, Shahjahan et al., 2020, Sharmin et al., 2016, Suchana et al., 2021, Shahjahan et al., 2021, Shahjahan et al., 2022). The current study revealed that VE provision in diet did not significantly influence the fish hematology. Similar results have been demonstrated in yellow catfish, *P. fulvidraco* (Lu et al., 2016). In contrast, VE significantly increased the Hb content and RBC count in largemouth bass, *M. salmoides* (Li et al., 2018). Shahkar et al., 2017 demonstrated that dietary VE enhance the Hb and WBCs while decreasing the BG level of Japanese eel, *Anguilla japonica*. In addition, dietary VE affected the hematocrit, Hb, RBCs and mean corpuscular hemoglobin (MCH) in rainbow trout, *O. mykiss* (Mohammad and Hosein, 2014). These findings may be resulted due to the variations in the efficiency of VE in the blood physiology of various species.

VE supplementation in diet did not bring any significant alteration in proximate composition of fish muscle in this study. Several studies reported the similar findings on several species such as European bass, *Dicentrarchus labra*, turbot, *Saltator maximus* and bream, *Megalobrama amblycephala* (Ruff et al., 2003; Zhang et al., 2017). However, Li et al., 2018, Lozano et al., 2017 reported that VE considerably improved protein levels in bass, *M. salmoides* and lowered the level of moisture in meager, *A. regius*, respectively.

Various studies revealed that formation of muscle fibers with small diameter surrounded by fibers having large diameter (Ahammad et al., 2021a, Ahammad et al., 2021b, Knutsen et al., 2019). A number of fish species commonly exhibited such types of muscle structure in various stages of their life cycle and these are generally known as hypertrophic and hyperplastic muscle growth (Aguiar et al., 2005, Asaduzzaman et al., 2019). For utmost of the cultured species, these types of muscle structure formation are considered as a common phenomenon throughout the life (Aguiar et al., 2005). In the current study, formation of hyperplastic muscle fiber may be resulted as a result of VE supplementation that was confirmed by existence of several immature fibers in Nile tilapia’s muscle. Hyperplasia has a great contribution in the development of fish muscle as well as growth. Thus development of new myotubes may be resulted due to the formation of new immature fiber that is covered by the comparatively large fiber (Dal Pai-Silva et al., 2003). This hyperplastic muscle growth due to VE supplemented diet may be attributed to the regulation of the associated growth-related genes by VE. However, in depth studies are needed to search out the specific trigger that controls the mechanisms of this type of muscle growth.

In summary, it is revealed that dietary VE plays an important role to enhance the production of Nile tilapia by improving the feed efficiency. Moreover, VE developed the morphological structure of fish muscle without hampering the fish hemato-biochemical indices. Further investigations are needed to determine its role at molecular level. Nonetheless, we should confirm the effects of VE on reproduction, immunity and stress management of the experimental species.

## Declaration of Competing Interest

The authors declare that they have no known competing financial interests or personal relationships that could have appeared to influence the work reported in this paper.
